# Randomized crossover trial of 2-week Garment electrocardiogram with dry textile electrode to reveal instances of post-ablation recurrence of atrial fibrillation underdiagnosed during 24-hour Holter monitoring

**DOI:** 10.1371/journal.pone.0281818

**Published:** 2023-02-24

**Authors:** Takeshi Machino, Kazutaka Aonuma, Kazushi Maruo, Yuki Komatsu, Hiro Yamasaki, Miyako Igarashi, Akihiko Nogami, Masaki Ieda

**Affiliations:** 1 Faculty of Medicine, Department of Cardiology, University of Tsukuba, Tsukuba, Japan; 2 Faculty of Medicine, Department of Clinical Research and Regional Innovation, University of Tsukuba, Tsukuba, Japan; 3 Faculty of Medicine, Department of Biostatistics, University of Tsukuba, Tsukuba, Japan; Prince Sattam Bin Abdulaziz University, College of Applied Medical Sciences, SAUDI ARABIA

## Abstract

**Background:**

Atrial fibrillation (AF) is the most common arrhythmia and often recurs despite catheter ablation. The recurrence of AF is often underdiagnosed by standard 24-hour electrocardiogram (ECG) because of its transient and silent nature. A garment-style ECG with a highly conductive textile electrode made of poly(3,4-ethylenedioxythiophene) poly(styrenesulfonate)(PEDOTPSS) and nanofiber (Garment ECG) has been developed that can provide longer-term continuous monitoring. This study investigated whether 2-week Garment ECG can reveal instances of AF recurrence in patients who are diagnosed as remaining in sinus rhythm by 24-hour Holter ECG.

**Methods:**

The open-label randomized crossover study enrolled 67 patients (63.1±10.6 years old, 53 men) who had undergone initial AF ablation. Three months after ablation, patients were randomly assigned to group 1 (n = 35), 2-week Garment ECG followed by 24-hour Holter ECG, or group 2 (n = 32), 24-hour Holter ECG followed by 2-week Garment ECG. The detection of AF recurrence was compared between the two devices.

**Results:**

The Garment ECG showed AF recurrence in 12 patients (18%) compared to 4 patients for the Holter ECG (6%, p = 0.008). The ECG acquisition rate was higher for Holter ECG than for Garment ECG (100.0% [interquartile range 100.0–100.0%] versus 82.4% [71.1–91.0%], p<0.001), but the Garment ECG provided longer total analysis time (11.0 days [9.0–12.2 days] for Garment; 1.0 day [1.0–1.0 day] for Holter, p<0.001).

**Conclusions:**

Despite the lower ECG acquisition rate, the 2-week Garment ECG revealed instances of AF recurrence after ablation in patients who were underdiagnosed by 24-hour Holter ECG.

**Trial registration:**

**Clinical Trial Registration:** URL: https://jrct.niph.go.jp/en-latest-detail/jRCTs032180018 Unique Identifier: jRCTs032180018.

## Introduction

Atrial fibrillation (AF) constitutes a major risk factor for cardiovascular events, cerebral infarction, dementia, and death [1,2] and is increasing rapidly as populations age around the world [3]. Catheter ablation is widely used to eliminate AF; however, approximately half of patients after AF ablation experience AF recurrence [4,5]. Such recurrence affects subsequent therapeutic choices for the use of repeated ablation and/or antiarrhythmic and anticoagulant agents, emphasizing the need for intensive AF detection.

The actual rate of AF detection depends on the duration of electrocardiogram (ECG) monitoring, but the current standard of care for post-ablation AF relies on 24-hour continuous monitoring using a conventional Holter ECG. About 50% of recurrent AF may be missed under current ECG monitoring for only 24 hours [6,7], because the transient episodes of recurrent AF that occur frequently after catheter ablation can be diagnosed only when they can be detected by ECG. Increasing the duration of ECG monitoring significantly increases AF detection [8].

Consumers have recently begun to use handheld devices connected to smartphone applications for self-diagnosis, but those devices tend to be unreliable and are impractical for use in a clinical setting [9]. Wearable ECG devices with adhesive gel electrodes are capable of recording for up to 14 days, but in actual clinical use the patches often fall off, and the practical wear-time is generally limited to about 7 days [10,11].

The recent development of a novel dry textile electrode (hitoe^®^, Toray Industries, Inc., Tokyo, Japan) may offer more extended recording options. This dry electrode, which is designed for Garment ECG applications and has been registered as a medical device in Japan (13B1X0001500034), is well-suited to recording ECGs in a non-invasive and continuous manner [12,13]. We conducted a prospective observational pilot study of AF recurrence in 18 patients who underwent ablation at our hospital, in which ambulatory ECG was obtained simultaneously from all patients, using both Garment ECG and Holter ECG, and data were automatically analyzed and compared between groups [14]. Results showed that Garment ECG and Holter ECG provided comparable rates of detection for AF and other arrhythmia events.

Based on these preliminary findings and previous studies in healthy participants [15,16], we designed the present study to further investigate the potential of this Garment ECG system with dry electrodes as a diagnostic tool for detecting AF recurrence after ablation. We used randomized crossover comparison to detect AF recurrence in 2-week Garment ECG compared with conventional 24-hour Holter ECG.

## Materials and methods

### Study design and participants

**Fig 1:**
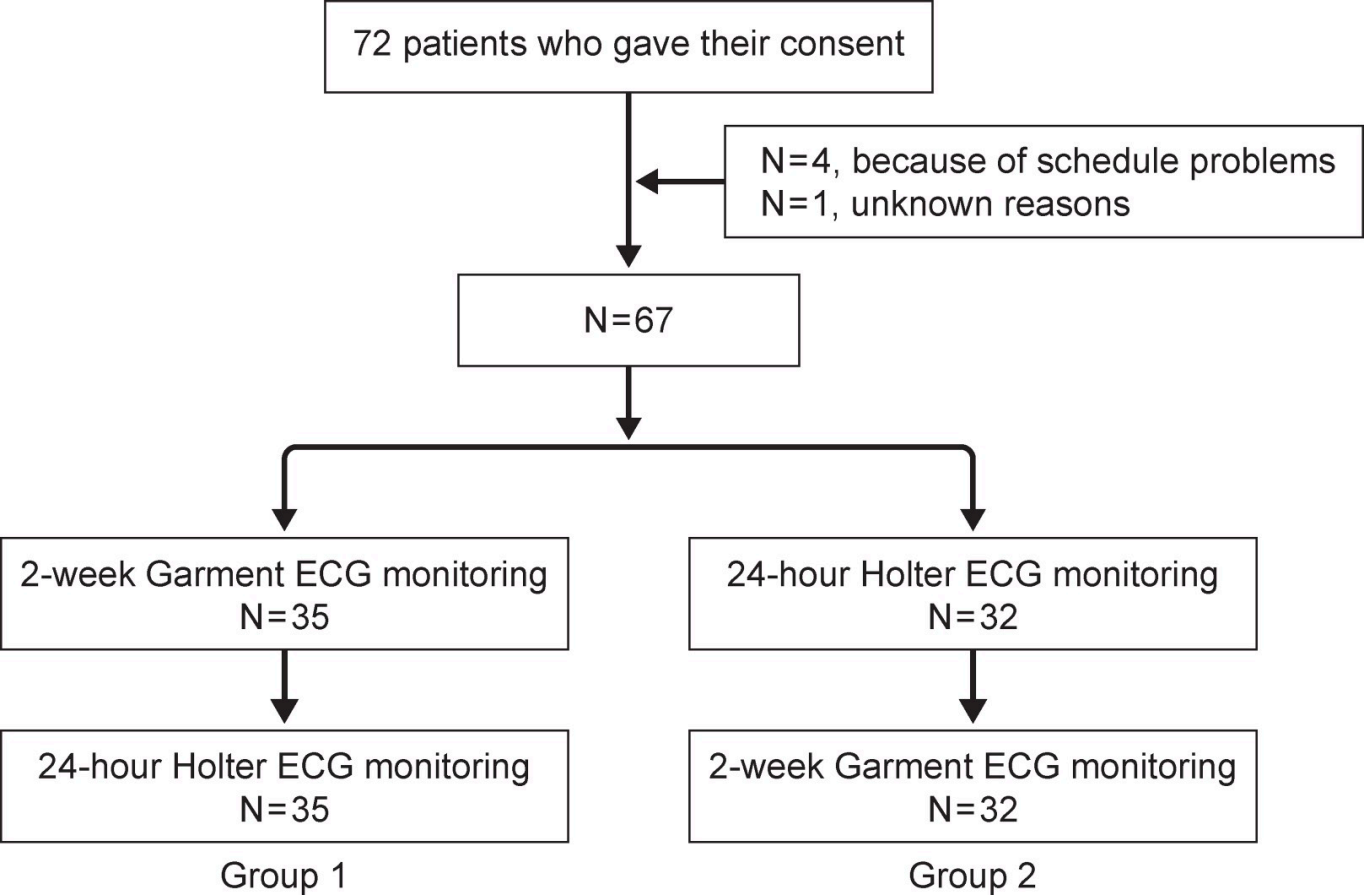
Patient flow diagram. ECG, electrocardiogram.

This was a randomized crossover comparative study with open design and block randomization (Fig 1). The crossover design provided uniform median duration of follow-up, allowing the comparison of two types of ECG monitoring for the two different wear-times of 1 day and 2 weeks. We applied block randomization to protect against potential bias, which under simple randomization could have been caused by a numerical imbalance in group assignment.

This study was registered in the Japan Registry of Clinical Trials (jRCT) as jRCTs 032180018. Patients provided written informed consent before enrollment. The protocol was approved by the University of Tsukuba Clinical Research Review Board, and the study was conducted in accordance with the Declaration of Helsinki and the Clinical Trials Act in Japan [17].

**Fig 2:**
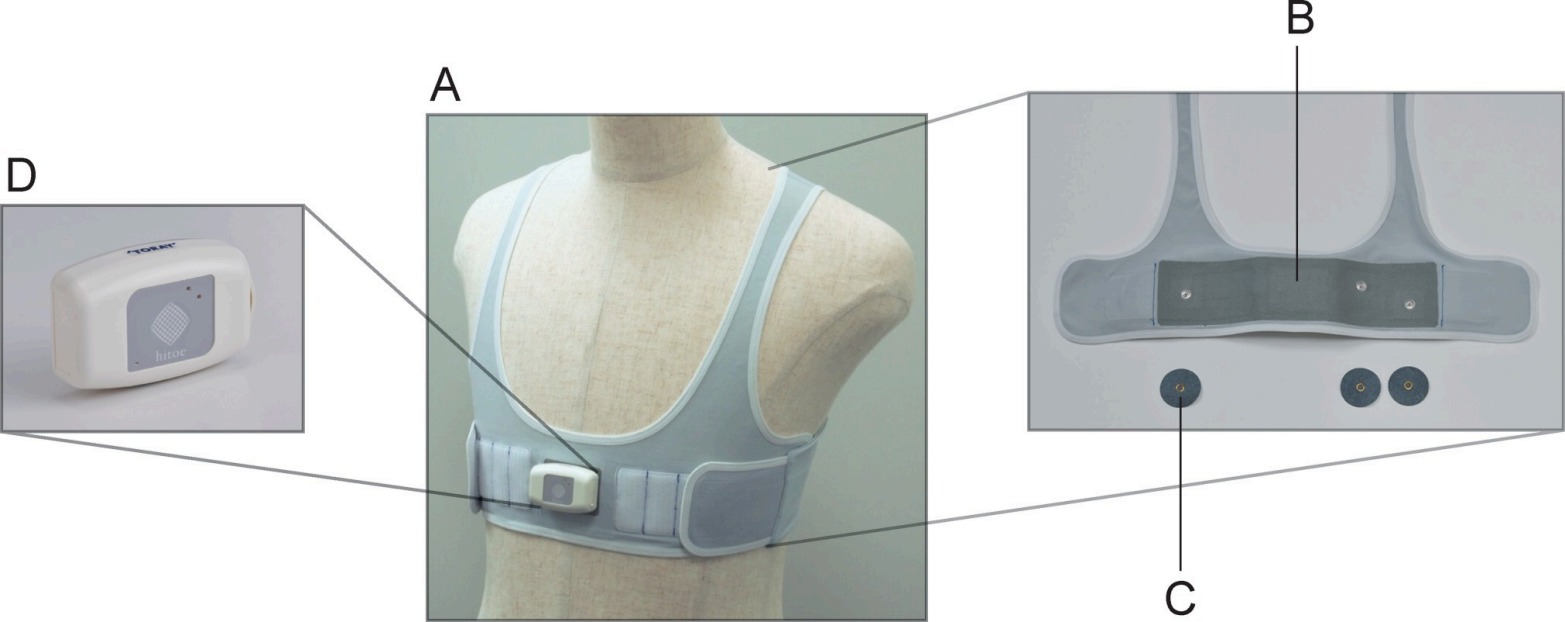
Garment ECG system with hitoe^®^ dry electrode. A, The hitoe^®^ dry electrode is sewn into a stretchy fabric used for sportswear, for a wearable ECG unit that can be washed. The unit can be put on and taken off easily by patients, facilitating near-continuous monitoring. B, The hitoe Medical Leads II: Lead wires are pre-installed on the wearable device and conduct the signal detected by the electrodes to the electrocardiograph. The pressure adjustment function allows the wearer to obtain the proper position and adherence, enabling comfortable extended monitoring. C, The hitoe Medical Electrode II: Electrodes are made of an electrically conductive textile material called hitoe. The polyester nanofiber fabric is coated with a dielectric polymer to prevent rash even with prolonged use. D, The hitoe ECG Recorder EV-301: The recorder is a compact lightweight Holter electrocardiograph, capable of continuous recording for up to 2 weeks without recharging. ECG, electrocardiogram.

This study was implemented at the University of Tsukuba Hospital. Eligible participants were 20 years of age or older at consent; had completed a first catheter ablation for AF; were scheduled for Holter ECG monitoring 3 months post-ablation at the University of Tsukuba Hospital; and consented to use a Garment ECG device (Fig 2) for 14 days and a Holter monitor for 24 hours. All participants had chest measurements (underbust where applicable) of 60–120 cm to accommodate the Small, Medium, and Large sizes available for the Garment device. Patients were excluded if they were prone to allergies or sensitive to adhesive tape; had a history of skin abnormalities such as redness, erosion, or scarring; or were using a cardiovascular implantable electronic device.

### Endpoints

The primary endpoint was the detection rate of patients with AF recurrence. AF recurrence was defined as AF or arterial flutter lasting 30 seconds or longer, a definition that is widely used for assessing AF after catheter ablation [7,14].

The major secondary endpoint was the ECG acquisition rate, which was defined as the ratio of “time for analysis (recording time − noise)” to total recording time. Recording time was defined as monitoring time minus the time for which monitoring was not possible because the patient had removed the garment for bathing, etc. Device comfort while wearing was assessed by questionnaire, and incidence of non-AF arrythmia events was also assessed.

Safety endpoints were presence of redness, erosion, or scarring. Before removing the investigational device, a clinical laboratory technologist and a clinical research coordinator reviewed the questionnaire and observed the site where the device was attached. If any injury to the skin was suspected, the site was examined by a physician.

### Procedures

After catheter ablation, patients provided informed consent to participate in this study. Those who met the inclusion criteria and did not meet the exclusion criteria were enrolled and randomly allocated to one of the two groups. Three months after catheter ablation, patients started ECG monitoring using the device that had been allocated to their group at randomization (2 weeks using the hitoe Medical Electrode II (Toray Medical Co., Ltd., Tokyo, Japan) with the hitoe ECG Recorder EV-301(Parama-Tech Co., Ltd., Fukuoka, Japan) and hitoe Medical Leads II (Toray Medical Co., Ltd., Tokyo, Japan) in the Garment ECG group or the disposable electrode NC-105CM (NIHON KOHDEN CORPORATION, Tokyo, Japan) with Long-term Recorder RAC-3103 (NIHON KOHDEN CORPORATION, Tokyo, Japan) and Patient Cables BJ-322D (NIHON KOHDEN CORPORATION, Tokyo, Japan) in the Holter group. At the end of the first time period, each patient was switched to the other device. The total for the two time periods was 15 days. Study staff reviewed all adverse reactions and undesirable events for each ECG device, and the patients completed questionnaires when they returned the last allocated ECG device. The ECG data were analyzed automatically using ECG analyzers: NEY-HEA3000 (Nexis Co., Ltd., Fukuoka, Japan) for the Garment ECG group and DSC-3300 (NIHON KOHDEN CORPORATION, Tokyo, Japan) for the Holter group. The results were entered into each patient’s electronic medical record. Patients were required to remove the Garment ECG device when bathing to protect the electrode from water, and in the event of magnetic resonance imaging because local heating from induced electromotive force might cause burn injuries. Use of topical agents for cutaneous pruritus was permitted.

Block randomization was processed, and a sequential number for ECG session was assigned automatically by a computer system, the UHCT ACReSS (University Hospital Clinical Trial Alliance Clinical Research Support System). Block size was ten, and the allocation ratio was equalized between the two groups. The allocation sequence was concealed through a third-party assignment implemented by the clinical research coordinators. Patients were enrolled by investigators other than the statistician.

### Statistical analysis

A previous study showed a 6.5% mean risk of an AF episode during a 1-day period and 16.7% mean risk of at least one AF episode during a 7-day period [18]. The occurrence of AF episodes each day was assumed to conform to a multivariate Bernoulli distribution using the Gaussian copula. We conservatively estimated a 5% daily risk of AF episode and hypothesized that 17.1% of patients would experience at least 1 episode during the 14-day period. The correlation coefficient between at least 1 episode during those 14 days and 1 episode during a 1-day period was assumed to be 0.460. Under these circumstances, we calculated that a minimum sample size of 68 patients would be required to obtain a power of 0.8 or higher for the main analysis. In the study, that number was set at 70 to allow for dropouts.

Efficacy analysis was conducted on the full analysis set (FAS), which included all patients who met the inclusion criteria but not the exclusion criteria. Patients who did not wear their assigned device after randomization and those for whom no data were available after randomization were excluded from the FAS. We used an exact McNemar’s test in the main analysis of the primary endpoint. In other analyses of the primary endpoint, the detection rate and exact confidence interval (CI) were calculated for each device. For the secondary endpoints, we applied the same analysis to binary variables as to the primary endpoint. Descriptive statistics (mean, standard deviation, median, and interquartile range) were calculated for frequency variables in both groups, and the Wilcoxon rank sum test was used to compare the groups. The significance level of the statistical test was set at 0.05, and a two-sided test was performed. The confidence interval was set at 95%. In dropouts, statistical tests were conducted for binary variables (yes/no) by imputing “No” if the data were missing for both devices or for one device and “No” for the other device, and by imputing “Yes” if the data were “Yes” for one device and missing for the other device. Imputation and modeling for missing values were not implemented for other analyses.

The safety analysis population included all patients except those for whom no data were available after randomization. The same analysis was performed as for the primary endpoint.

All analyses were performed using SAS ver. 9.4 (SAS Institute Inc., Cary, NC, USA).

## Results

Patients were registered at the University of Tsukuba Hospital from August 2018 to December 2019. The follow-up period ended in April 2020.

### Study population

Of the 72 patients who consented to participate, 67 completed the study, 4 withdrew because of scheduling problems, and 1 withdrew without providing a reason. The patients were randomly assigned to group 1 (n = 35), which started the study using the Garment ECG with textile electrode for 2 weeks and then the Holter ECG with gel electrode for 24 hours, or group 2 (n = 32), which started the study by wearing the Holter monitor for 24 hours and then the Garment ECG for 2 weeks. Data yielded 67 paired ECGs (2 from each patient) from the Garment ECG for 2 weeks and the Holter ECG for 24 hours (Fig 1).

### Patient background

The mean age was 63.1±10.6 years old, and 53 (79%) were men. There were 46 patients (69%) with paroxysmal AF, 18 (27%) with persistent AF, and 3 (4%) with long-standing persistent AF. Underbust circumference averaged 92.0±9.1 cm, appropriate for the available sizes (Small, Medium, and Large) of the Garment ECG. Most patients received anticoagulation therapy, and some were taking antiarrhythmics before and 3 months after ablation (Table 1).

**Table 1:** Patient characteristics.

	All (n = 67)	Group 1 Garment-first group (n = 35)	Group 2 Holter-first group (n = 32)
Age, mean(SD), y	63.1(10.6)	66.8(9.1)	59.2(10.7)
Male, n(%)	53(79)	30(86)	23(72)
Underbust, mean(SD), cm	92.0(9.1)	93.7(7.9)	90.1(10.1)
Height, mean(SD), cm	166.6(9.3)	166.4(8.3)	166.7(10.4)
Weight, mean(SD), kg	67.3(11.2)	68.5(10.0)	66.1(12.4)
EF, mean(SD), %	63.1(6.8)	63.0(6.0)	63.3(7.8)
LAD, mean(SD), mm	37.8(6.8)	38.3(6.5)	37.2(7.1)
LAVI, mean(SD), mL/m^2^	34.1(11.6)	34.9(12.1)	33.3(11.0)
BNP, mean(SD), pg/mL	108.4(147.8)	112.7(130.1)	103.8(167.0)
eGFR, mean(SD), mL/min/1.73m^2^	71.4(18.7)	68.6(20.8)	74.5(15.9)
Hemoglobin, mean(SD), g/dL	14.2(1.7)	14.2(1.9)	14.2(1.6)
Wear size, n(%) Large/Medium/Small	16(24)/45(67)/6(9)	9(26)/25(71)/1(3)	7(22)/20(63)/5(16)
PVI method, n(%) Cryo-balloon ablation Hot-balloon ablation Laser-balloon ablation Radiofrequency ablation	25(37) 8(12) 2(3) 32(48)	13(37) 4(11) 2(6) 16(46)	12(38) 4(13) 0(0) 16(50)
Heart failure, n(%)	4(6)	2(6)	2(6)
Hypertension, n(%)	39(58)	22(63)	17(53)
Diabetes mellitus, n(%)	10(15)	4(11)	6(19)
Vascular disease, n(%)	1(1)	0(0)	1(3)
AF type, n(%) Paroxysmal AF Persistent AF Long-standing persistent AF	46(69) 18(27) 3(4)	26(74) 9(26) 0(0)	20(63) 9(28) 3(9)
CHADS_2_ score, n(%) 0 1 2 3	24(36) 27(40) 13(19) 3(4)	10(29) 18(51) 4(11) 3(9)	14(44) 9(28) 9(28) 0(0)
CHA_2_DS_2_ VASc score, n(%) 0 1 2 3 4 5	13(19) 16(24) 22(33) 13(19) 2(3) 1(1)	4(11) 10(29) 13(37) 6(17) 1(3) 1(3)	9(28) 6(19) 9(28) 7(22) 1(3) 0(0)
Oral anticoagulant, n(%) Before ablation Rivaroxaban Apixaban Dabigatran etexilate Edoxaban tosilate hydrate Warfarin potassium None 3 mo after ablation Rivaroxaban Apixaban Dabigatran etexilate Edoxaban tosilate hydrate Warfarin potassium None	7(10) 10(15) 15(22) 29(43) 4(6) 2(3) 8(12) 11(16) 11(16) 25(37) 4(6) 8(12)	4(11) 4(11) 7(20) 15(43) 3(9) 2(6) 5(14) 4(11) 6(17) 12(34) 3(9) 5(14)	3(9) 6(19) 8(25) 14(44) 1(3) 0(0) 3(9) 7(22) 5(16) 13(41) 1(3) 3(9)
Antiplatelet, n(%) Before ablation 3 mo after ablation	3(4) 2(3)	1(3) 0(0)	2(6) 2(6)
Class Ⅰantiarrhythmic drug, n(%) Before ablation 3 mo after ablation	16(24) 6(9)	7(20) 4 (11)	9(28) 2 (6)
Amiodarone, n(%) Before ablation 3 mo after ablation	5(7) 4(6)	1(3) 2(6)	4(13) 2(6)
Bepridil, n(%) Before ablation 3 mo after ablation	4(6) 2(3)	2(6) 1(3)	2(6) 1(3)
Beta blocker, n(%) Before ablation 3 mo after ablation	28(42) 21(31)	12(34) 10(29)	16(50) 11(34)
Ca antagonist, n(%) Before ablation 3 mo after ablation	18(27) 11(16)	12(34) 7(20)	6(19) 4(13)
Digitalis, n(%) Before ablation 3 mo after ablation	1(1) 0(0)	0(0) 0(0)	1(3) 0(0)

AF, atrial fibrillation; BNP, B-type natriuretic peptide; EF, ejection fraction; eGFR, estimated glomerular filtration rate; LAD, left anterior descending artery; LAVI, left atrial volume index; PVI, pulmonary vein isolation; SD, standard deviation.

### Efficacy

**Fig 3:**
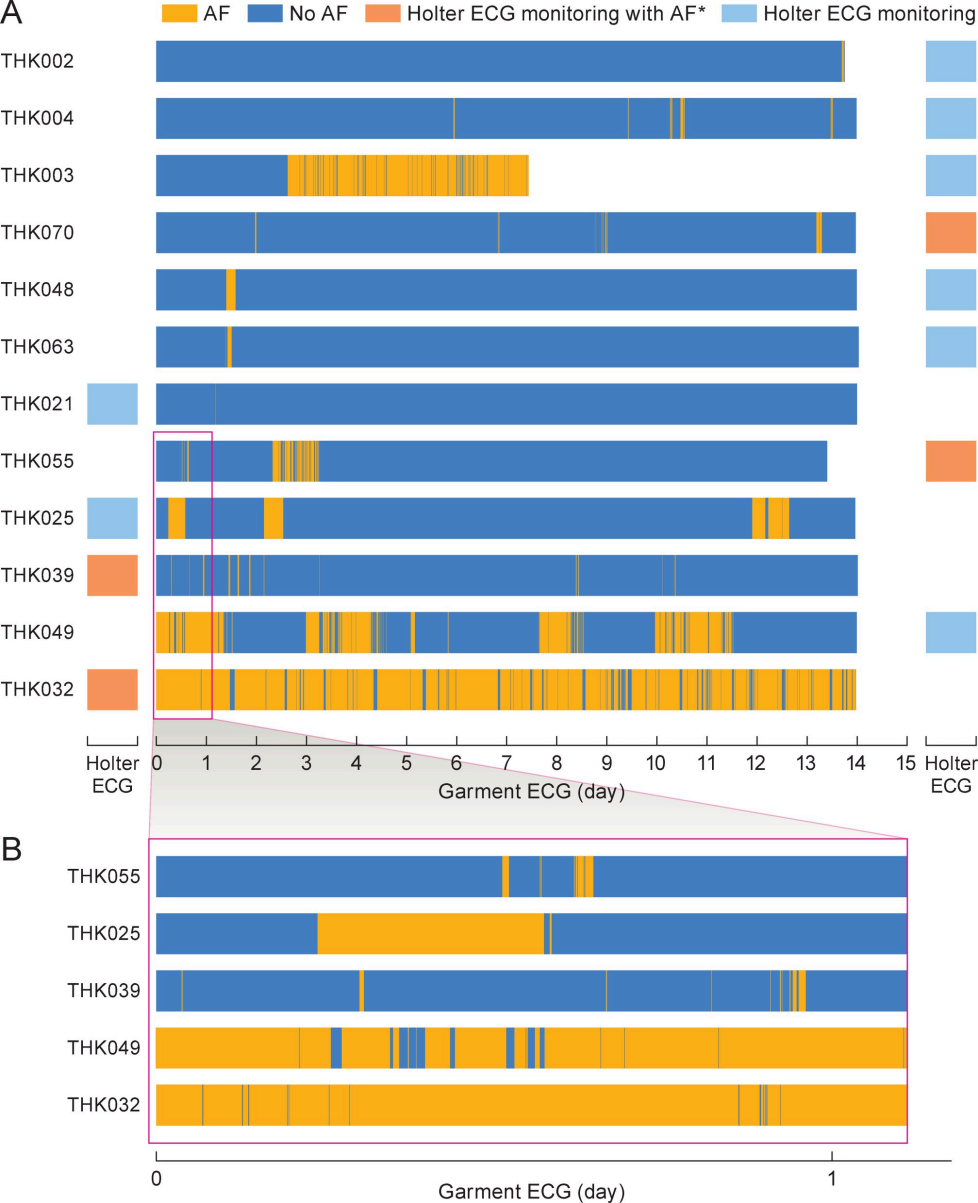
AF occurrences during the follow-up period. A, AF occurrences in 12 relapsed patients. B, Data from 5 patients who had AF on the first day of Garment ECG measurements. * Of the 12 patients, 4 also showed AF on the Holter 24-hour ECG. AF, atrial fibrillation; ECG, electrocardiogram.

**Fig 4:**
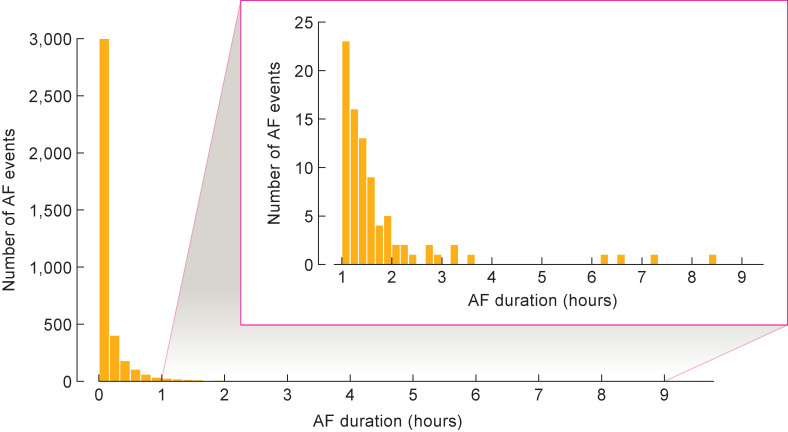
AF duration in the Garment ECG measurement period. AF, atrial fibrillation; ECG, electrocardiogram.

The 2-week Garment ECG monitoring detected 12 patients (18%) with AF recurrence, of whom only 4 were detected by 24-hour Holter ECG (6%, p = 0.008) (Table 2). In those 12 patients, the Garment ECG showed that AF recurred at non-uniform intervals with various burden during the 14 days of monitoring, including at the very end of the monitoring period in 1 patient (Fig 3A). In our study, 4 of 12 patients with recurrent AF were symptomatic (2 cases of palpitation and 1 case each of dizziness and shortness of breath). In 5 of the 8 asymptomatic patients, AF was detected only during the 2-week Garment ECG. A magnified view of the first monitoring day shows early and short AF recurrences in detail (Fig 3B). The recorded duration of AF ranged widely, from 30 seconds to 8 hours 23 minutes, with the majority of AFs being short (Fig 4).

**Table 2:** Primary and secondary endpoint results.

	Garment ECG (n = 67)	Holter ECG (n = 67)	P value
Detection rate for AF* AF recurrence, n(%)	12(18)	4(6)	0.008^†^
ECG acquisition^‡^^§^ Acquisition rate, % Total analysis time, days	82.4(71.1–91.0) 11.0(9.1–12.2)	100.0(100.0–100.0) 1.0(1.0–1.0)	<0.001^||^ <0.001^||^
Arrhythmia events other than AF^‡^ Incidence of PACs, % Incidence of PVCs, %	0.09(0.03–0.33) 0.00(0.00–0.05)	0.09(0.03–0.48) 0.00(0.00–0.05)	0.014^||^ 0. 980^||^

*AF recurrence defined as detection of AF continuing for ≥30 seconds.

^†^P values based on McNemar test.

^‡^Data are presented as median and interquartile range.

^§^ECG acquisition rate is presented as (Total duration recorded–Duration of noise recorded) / Total duration recorded.

^||^P values based on Wilcoxon signed rank test.

AF, atrial fibrillation; ECG, electrocardiogram; PACs, premature atrial contractions; PVCs, premature ventricular contractions.

The ECG acquisition rate was 100.0% (interquartile range [IQR] 100.0–100.0%) for Holter and 82.4% (71.1–91.0%) Garment ECG, significantly higher for the Holter monitoring (p<0.001). However, the mean total analysis time was considerably longer for the Garment ECG than for Holter monitoring (11.0 days [9.1–12.2 days] versus 1.0 day [1.0–1.0 day], p<0.001) (Table 2). Questionnaire results showed that wearing comfort was generally better for the 2-week Garment ECG (79%) than for 24-hour Holter ECG (7%), with only 13% of patients reporting no difference between the two devices (Table 3).

**Table 3:** Results from questionnaire on wearability of the garment ECG device (secondary endpoint).

Question	n(%)
Was the unit difficult to put on? Very difficult Somewhat difficult Easy Very easy	0(0) 14(21) 41(61) 12(18)
Was the unit difficult to take off? Very difficult Somewhat difficult Easy Very easy	1(1) 3(4) 37(55) 26(39)
How did the fabric feel to the touch? Very uncomfortable Somewhat uncomfortable Quite comfortable Very comfortable	1(1) 9(13) 40(60) 17(25)
How was the tightness of the device? Didn’t want to wear it for more than a day Okay to wear for 2–3 days Okay to wear for a week Okay to wear for 2 weeks	2(3) 13(19) 23(34) 29(43)
How was the wearability compared to your previous experiences with a Holter monitor? Previous experiences were better About the same The Garment device was better	5(7) 9(13) 53(79)
How was the design of the Garment device? Bad Rather bad Good Very good	0(0) 9(13) 48(72) 10(15)

The Holter 24-hour ECG demonstrated a slightly higher frequency of premature atrial contractions (PACs) than the 2-week Garment ECG (0.09% [IQR 0.03–0.48%] versus 0.09% [0.03–0.33%], p = 0.014) (Table 2).

### Safety

There were no serious adverse events or unexpected adverse events with either monitoring system. The expected effects on the skin were: redness (n = 2, 3%), erosion (n = 0, 0%), and scar (n = 0, 0%) for 2-week Garment ECG; and redness (n = 0, 0%), erosion (n = 0, 0%), and scar (n = 0, 0%) for 24-hour Holter ECG.

## Discussion

A Garment ECG using hitoe^®^ dry electrodes is currently in use in Japan as a medical device for non-invasive continuous ECG monitoring. Although no long-term prospective studies have yet been conducted to investigate usefulness, this type of monitoring appears particularly promising in areas such as detecting AF recurrence after ablation. In this crossover study, the efficacy of 2-week Garment ECG was analyzed for a median of 11 days. The Garment ECG showed recurrent AF after ablation at three times greater with a higher AF burden than the conventional 24-hour Holter ECG, a statistically significant difference.

Long-term ECG monitoring options for AF are being explored using implantable devices and non-invasive external recorders. However, implantable devices are expensive and not covered by health insurance, and non-invasive recorders often provide false positives or are otherwise unreliable [9,19,20]. Wearable patch-type devices with embedded adhesive gel electrodes are non-invasive, relatively inexpensive, and capable of recording for up to 14 days, but the gel electrode becomes unusable if the patch peels off, which generally limits use to 7 days or less [10,11]. In particular, each peeled-off patch requires the patient to visit the clinic for a replacement, increasing the burdens of inconvenience and cost and reducing the feasibility of gel electrodes for extended outpatient use. In contrast, the Garment ECG using hitoe^®^ dry electrodes can provide at least 2 weeks of reliable long-term monitoring in an actual clinical setting.

In the present study the ECG acquisition rate for the Garment ECG was 82.4%, with noise accounting for the difference between the actual acquisition rate and 100.0%. However, even at this noise level, results from our study support the clinical usefulness of this 2-week wearable system. Because the system is constructed in the form of a garment, there are some concerns that the electrode might shift with body movement, which could increase noise. Our pilot study showed a slightly higher ratio of noise signals than for the 24-hour Holter ECG, but the noise signals did not affect heart rate and were thus considered not to be clinically important [14]. Notably, participants in the pilot study were monitored in-hospital while the present study was conducted exclusively in outpatients. This difference could account for the higher level of noise and the ECG acquisition rate of 82.4% in our study. In this regard, earlier research reported up to 35% higher noise levels during movement for wearables than for Holter monitors [15]. These findings suggest that this monitoring method is reliable and particularly amenable to routine clinical use after AF ablation.

Garment ECG monitoring showed evidence of AF recurrence among 12 patients at non-uniform intervals during the 2 weeks of monitoring, including 1 patient whose first recurrence occurred at the very end of the monitoring period. This suggests that AF recurrence may be underdiagnosed by Holter ECG monitoring for two reasons: because post-ablation AF occurs only rarely in some patients, and also because the timing of those occurrences is highly non-uniform. In addition, 8 of 12 patients were asymptomatic in this study, supporting previous findings that post-ablation AF recurrence is frequently asymptomatic and can only be diagnosed from ECG recordings [5,21]. Post-ablation AF can also occur sporadically (Fig 3) and is often of short duration (Fig 4). These findings underline the usefulness of continuous post-ablation monitoring for at least 2 weeks.

The incidence of AF may vary depending on the patient population, and long-term monitoring will be especially beneficial in patients with lower AF incidence. The Garment ECG can support even more prolonged monitoring in the future by providing improved longer-term data storage and higher-capacity batteries while continuing to eliminate the risk of accidental patch removal. This system thus may prove more beneficial than wearable patch-type devices, not only in post-ablation patients but also in a wider population of patients who need access to this type of monitoring.

Because PACs can trigger AF, we also assessed the incidence of PACs using Garment and Holter ECGs. The difference in results was statistically significant but had no clinical significance, showing the same median value of 0.09% for both devices.

While patch-type ECG monitors with gel-type electrodes have been associated with mild skin irritation in some patients [22,23], we found no evidence of skin irritation with the Garment ECG monitoring system using hitoe^®^ dry electrodes. In a brief questionnaire, the participating patients indicated that the Garment ECG device was more comfortable than the 24-hour Holter monitor. These results were some of the factors that enabled patients to wear the device continuously for 2 weeks.

One limitation of this study was the fact that, because the Garment ECG monitoring system with a dry electrode is not waterproof, AF monitoring time was interrupted when the patients removed the garment to bathe. Another limitation was the small sample size of this study, with only 12 out of 67 patients experiencing recurrent AF after ablation. Although we found a significant difference in the detection of post-ablation AF events between the 2 groups, further studies in a larger population are warranted. In assessing the risk of bias in this study [24], we should first consider the effects of assignment to intervention. Although researchers and participants were aware of group assignment, there were no deviations from the intended intervention, and automated AF detection reduced the risk of assessment bias. Furthermore, there were no failures in implementing the intervention and no instances of non-adherence to the assigned intervention regimen, which reduced the risk of adherence bias even though the study staff also knew to which group each participant was assigned. Data on the primary endpoint of recurrent AF were obtained from all participants, and there were no missing data, which reduced the risk of outcome bias. Finally, the use of a crossover design reduced the risk of measurement bias from differences in the median duration of follow-up between the two groups.

In conclusion, continuous 2-week recording with a garment-style wearable ECG monitor equipped with a textile electrode showed a higher extent of AF recurrence than with a Holter 24-hour ECG monitor. The 2-week monitoring period more than compensated for the lower acquisition rate of the Garment ECG system, revealing a higher level of hidden AF recurrence than was recorded with the Holter 24-hour ECG.

## Supporting information

S1 Checklist CONSORT 2010 checklist of information to include when reporting a randomised trial*. (DOC)

S1 File Study protocol. (DOCX)

## Abbreviations

AF: Atrial fibrillation
CI: confidence interval
ECG: electrocardiogram
FAS: full analysis set
ILR: implantable loop recorder
IQR: interquartile range
jRCT: Japan Registry of Clinical Trials
PEDOTPSS: poly(3,4-ethylenedioxythiophene) poly(styrenesulfonate)
UHCT: ACReSS, University Hospital Clinical Trial Alliance Clinical Research Support System)
