# Supplementary material for: Randomized crossover trial of 2-week Garment electrocardiogram with dry textile electrode to reveal instances of post-ablation recurrence of atrial fibrillation underdiagnosed during 24-hour Holter monitoring
Source: PLoS One. 2023 Feb 24;18(2):e0281818. doi: 10.1371/journal.pone.0281818 (PMC9955627; doi:10.1371/journal.pone.0281818)
Supplement: S1 File — (DOCX) [file pone.0281818.s002.docx]

Crossover Randomized Controlled Trial of Garment-type Two-week Electrocardiogram Monitoring System vs. Conventional 24-hour Electrocardiogram Monitoring System for Diagnosis of Post-ablation Recurrence of Atrial Fibrillation (hitoe-2)

Investigator

Name: Takeshi Machino, MD, PhD

Affiliation: Department of Cardiology, Faculty of Medicine, University of Tsukuba

E-mail: [machino@md.tsukuba.ac.jp](mailto:machino@md.tsukuba.ac.jp)

Emergency contact: +81-29-853-3143

Planned study period: August 1, 2018 to December 31, 2020

| Draft protocol version No. | Date of preparation |
| --- | --- |
| 0.1 | April 10, 2018 |
| 0.2 | April 29, 2018 |
| 0.3 | May 16, 2018 |
| 0.4 | June 28, 2018 |
| 0.5 | June 29, 2018 |
| 0.6 | July 4, 2018 |
| 0.7 | July 17, 2018 |
| 1.0 | January 29, 2019 |
| 1.1 | January 6, 2020 |

<Table of Contents>

1. Administrative Structure
2. Background
3. Objectives
4. Description
5. Inclusion, Exclusion, and Withdrawal Criteria
6. Intervention
7. Efficacy Evaluation
8. Safety Evaluation
9. Statistical Design
10. Source Data Verification
11. Quality Control and Quality Assurance
12. Ethical Considerations
13. Record Management
14. Payments and Compensations
15. Publication of Information on Clinical Study
16. Study Period
17. Informed Consent
18. Requirements for Proper Conduct of Clinical Study
19. List of References/Literature
20. Appendices (package inserts)
21. **Administrative Structure**
    1. Investigator

Takeshi Machino (Lecturer, Department of Cardiology, Faculty of Medicine, University of Tsukuba, Ibaraki, Japan; Tel +81-29-853-3143)

- 1. Study Site Address and Contact Information

University of Tsukuba Hospital, 2-1-1 Amakubo, Tsukuba, Ibaraki, Japan (Tel: +81-29-853-3143)

- 1. Data Management Director and Staff

Director: Takeshi Machino (Lecturer, Department of Cardiology, Faculty of Medicine, University of Tsukuba, Ibaraki, Japan; Tel +81-29-853-3143)

Staff: Yoshihide Udagawa (Manager, CMIC HealthCare Institute Co., Ltd., Tokyo, Japan; Tel +81-3-6779-8164)

- 1. Monitoring Director

Saori Shin (Chief, R & D Department, U-NEXT Co., Ltd., Fukuoka, Japan; Tel +81-92-415-1156)

- 1. Audit Director

Shinichiro Ueda (Head of Clinical Research Education and Management Center, University of the Ryukyus Hospital, Okinawa, Japan; Tel +81-98-895-1508)

- 1. Statistical Analysis Staff

Kazushi Maruo (Associate Professor, Department of Biostatistics, Faculty of Medicine, University of Tsukuba Ibaraki, Japan; Tel +81-29-853-3914)

1. **Background**

Atrial fibrillation (AF) is a major risk factor for all-cause death, cardiovascular death, and cerebral infarction, and is becoming much more prevalent as the population is aging. As catheter ablation has become widely used to treat AF and the presence or absence of post-ablation AF recurrence greatly affects decision of subsequent treatment policy, including re-ablation and medication with an antiarrhythmic drug, anticoagulant, etc., the development of technology that detects AF with certainty and without omission is a critical challenge.

While AF detection rate depends on the duration of electrocardiogram (ECG) monitoring, the current standard of post-ablation care involves Holter ECG, which allows only 24-hour monitoring. In particular, asymptomatic AF, which is reportedly common after ablation, cannot be diagnosed unless recorded on ECG, as the possibility has been pointed out that approximately half of recurrent AF cases may be missed^1, 2^. An implantable loop recorder (ILR), which requires subcutaneous implantation surgery, provides long-term continuous ECG monitoring and thereby enables detection of even infrequent AF. However, this approach is invasive, and is not covered by the Japanese National Health Insurance, except for cerebral infarction and syncope of unknown cause. Non-invasive ECG using external loop recorder with built-in algorithm for automated detection of arrhythmia has been reported to be effective for AF detection^3, 4^, but has, on the other hand, been associated with a problem of high false-positive rate compared with conventional Holter ECG, due to poor ECG quality and algorithm performance^5^.

The garment-type ECG monitoring system using a dry electrode (hitoe^®^), newly developed by Toray Industries, Inc. (Toray), enables non-invasive 2-week continuous ECG monitoring. In fiscal year 2017, a study was performed at the Department of Cardiology of University of Tsukuba hospital to compare this system with Holter ECG using the conventional gel electrodes in concurrent ECG monitoring in 18 patients who underwent ablation treatment for AF (H29-88). Comparison of the results of automatic analysis of ECG data obtained using the dry electrodes (hitoe^®^) and those using the gel electrodes demonstrated that both types of electrodes had comparable detection rates of various types of arrhythmia, including AF episodes differing in duration, etc. Since this suggests that the garment-type 2-week ECG monitoring system using the dry electrodes (hitoe^®^) is likely to offer a highly useful tool for diagnosis of post-ablation AF recurrence, the present study was planned. In addition, the garment-type ECG monitoring system, which can be easily put on and taken off, allows bathing during the monitoring period, and is also expected to reduce skin symptoms, as those associated with conventional adhesive gel electrodes, through the employment of the dry electrodes (hitoe®), which are made of highly hydrophilic polyester nanofiber.

1. **Objectives**

The objective of the study is to investigate, in a randomized crossover design, whether the garment-type 2-week Holter ECG monitoring system provides higher AF detection rate compared to the conventional 24-hour Holter ECG monitoring system for diagnosis of post-ablation AF recurrence.

1. **Description**
   1. Endpoints
      1. Primary endpoint

Detection rate of patients with recurrent AF (atrial fibrillation lasting at least 30 seconds)

- - 1. Secondary endpoints
- ECG acquisition rate: (total recording time – noise recording time)/total recording time
- Device comfort while wearing (questionnaire: attachment)
- Incidence of non-AF arrhythmia events
  1. Study Design

Open-label, crossover, block-randomized controlled trial

- 1. Study Schema (flowchart)


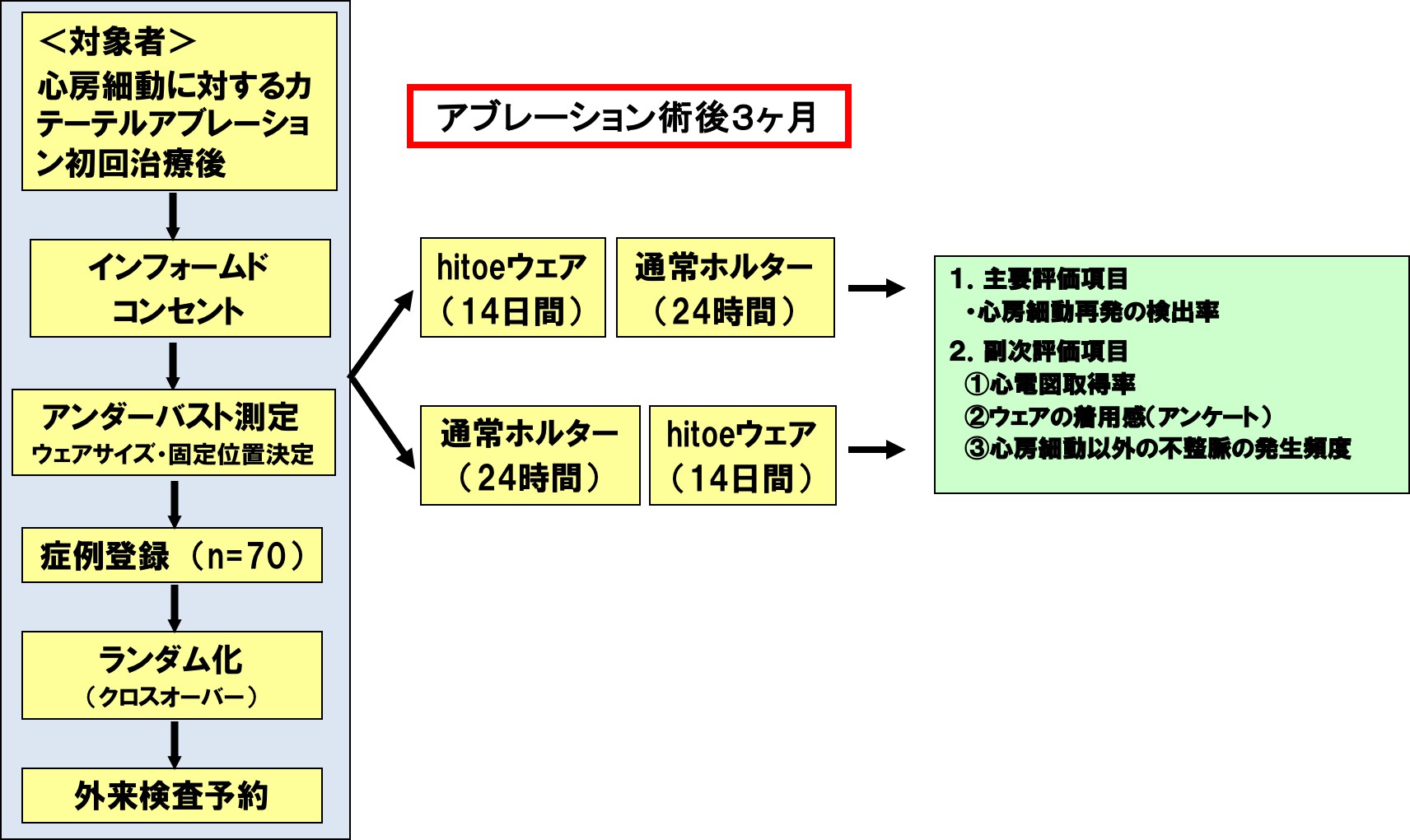


1. Primary endpoint

🞄 Recurrent AF detection rate

2. Secondary endpoints

i. ECG acquisition rate

ii. Wearing comfort (questionnaire)

iii. Incidence of non-AF arrhythmia

Three months post-ablation

hitoe wear

(14 days)

hitoe wear

(14 days)

Conventional Holter

(24 hours)

Conventional Holter

(24 hours)

<Potential participants>

Patients who underwent initial catheter ablation for AF

Informed consent

Underbust measurement

Wear size/fixation determination

Patient enrollment (n = 70)

Randomization

(crossover)

Appointment for outpatient ECG monitoring

- 1. Description of Study Devices
     1. Data on study devices

Information on the study devices used is presented in the table below.

| Equipment used | Continuous ECG monitoring with medical hitoe | Conventional Holter ECG monitoring |
| --- | --- | --- |
| Single-use ECG electrode | hitoe Medical electrode Ⅱ  (Registration No.: 13B1X00015000034) | Disposable electrode for ECG monitoring NC-105CM  (Registration No.: 13B1X00206000191) |
| ECG cable and lead wire | hitoe Medical lead Ⅱ  (Registration No.: 13B1X00015000035) | Lead cord BJ-322D  (Registration No.: 13B1X00206000065) |
| Holter ECG | hitoe ECG system EV-301  (Certification No.: 230AFBZX00014000) | Long-term ECG recorder RAC-3103  (Approval No.: 21400BZZ00285000) |
| Analysis software | ECG analyzer NEY-HEA3000  (Certification No.: 228AGBZX00099000) | Long-term ECG analyzer DSC-3300  (Approval No.: 21200BZZ00662000) |
| Other | Dedicated wear (non-medical device) | - |

For details of each device, see its package insert. For how to wear the garment-type device, see separate sheet “How to Wear the Device.”

| 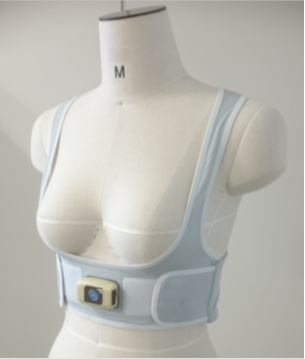 | 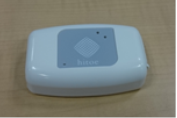 | 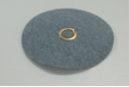 |
| --- | --- | --- |
| **ECG monitoring wear (hitoe® wear)** | **ECG recorder** | **ECG electrode** |

- - 1. Expected adverse reactions and expected defects

Refer to the package insert of each device. In particular, long-term use of the garment-type device may cause redness, skin erosion, and rarely scarring at the device contact area in patients with sensitive skin (with a predisposition to allergy).

- - 1. Wear and electrode use experience

A 48-hour patch test of the electrodes has been performed in healthy subjects to ensure that there are no associated skin disorders. A type of electrode-build-in wear has been commercially available under the brand name of "C3fit IN-pulse (IN-pulse)" for sports use since December 2014, and has been used as heart-rate monitoring wear for "Worker Watch Service" since August 2016. Furthermore, it has been under development as heart-rate monitoring wear for rehabilitation use at Fujita Health University since February 2017, and has been used by 41 patients for 207 person-days without any evidence of occurrence of adverse events.

- 1. Planned Duration of Patient Participant and Follow-up Period

After catheter ablation, informed consent will be obtained from potential participants. Those who satisfy all of the inclusion criteria and none of the exclusion criteria will be enrolled in the study, and an appointment for long-term ECG monitoring 3 months post-ablation will be arranged for them. Patients will undergo two different types of long-term ECG monitoring for a total of 15 days according to the assigned sequence and will be asked to answer a questionnaire when returning their ECG devices and observed for occurrence of adverse event, at the end of their participation in the study.

- 1. Study Termination, Discontinuation, and Interruption
     1. Study termination

At the end of the study, the investigator will publish the results in the Japan Registry of Clinical Trials (jRCT) by promptly submitting a notification of study termination to the Minister of Health, Labour and Welfare. The termination notification shall be prepared using Attached Form 1, “Notification of Trial Termination Notice," available on the website of the Ministry of Health, Labour and Welfare (MHLW).

- - 1. Study discontinuation/interruption
- The investigator shall consider whether to continue the study or not if any of the following are satisfied:

1) Any significant information regarding the quality, safety, or efficacy of the study devices becomes available

- - - 1. Any fact or information that compromises the properness or reliability of the study conduct becomes available
      2. It is judged difficult to recruit sufficient number of patients to achieve the planned sample size
      3. The study objectives are achieved prior to achievement of the planned sample size or planned duration, or it is judged that expected results cannot be obtained
      4. The certified review board (CRB) requests that the study protocol, etc. be changed and it is judged difficult to accept the request
    1. Policy of measures to be taken for patients after study discontinuation/termination

ECG monitoring may be performed after study discontinuation or termination at the discretion of the treating physician or at the patient's request.

- Upon decision to discontinue the clinical study, the investigator shall take the following actions:

1. Notify the CRB and the Minister of Health, Labour and Welfare within 10 days after the day of discontinuation.
2. Take appropriate actions for applicable patients. Solicit the opinions of the CRB with regard to when and how the study should be terminated in association with the measures for applicable patients, as necessary. After submission of the discontinuation notification, continue to make reports of adverse events and periodic reports, etc. until the termination of the clinical study.
3. The termination of clinical study after discontinuation refers to the time when the measures for applicable patients have been completed and the study is terminated.
4. After submission of the discontinuation notice and completion of the measures for applicable patients, submit a clinical study report for each protocol within one year, as a rule, from the day of discontinuation or the day of the end of the period for collecting data on all endpoints, whichever is later.
5. Include the presence or absence of patients required to be followed up in the discontinuation notification.
6. After submission of the discontinuation notification, if there are any changes to matters regarding the progress of the clinical study, continue to report amendments of the protocol until the clinical study is terminated.
   1. Procedures for Study Device Maintenance and Supply

The study devices will be provided by Toray and stored in the University of Tsukuba Hospital Functional Testing Laboratory (responsible person: Noriko Iida). Malfunctioning units shall be collected by Toray and replaced with new supplies, as necessary.

- 1. Procedures for Patient Enrollment and Assignment

When registered in the EDC (ACReSS), patients will be automatically assigned by block randomization to either sequence of ECG monitoring (hitoe^®^ wear first or conventional Holter first).

- 1. Specification of Raw Sources

1. Medical records (electronic medical records)
2. Informed consent forms (ICFs) (scanned into electronic medical records and stored in a medical record archive)
3. ECG results (captured in electronic medical records)
4. Questionnaires (scanned into electronic medical records and stored in a medical record archive)
5. **Inclusion, Exclusion, and Withdrawal Criteria**

Patients who satisfy all of the inclusion criteria below and none of the exclusion criteria below will be eligible for enrollment in the study.

- 1. Inclusion Criteria

1. Underwent the initial catheter ablation for AF
2. Age ≥ 20 years at the time of informed consent
3. Holter ECG is scheduled 3 months after ablation at University of Tsukuba Hospital
4. Consented to 14-day wearing of the garment-type ECG system
5. Underbust size in the range of 60 to 120 cm (3 wear sizes: S, M, and L available)
   1. Exclusion Criteria
6. Predisposition to allergy
7. History of such skin conditions as redness, erosion, and scarring due to adhesive sensitivity
8. Use of a pacemaker or electrical defibrillator

(This is set because a pacing pulse detector is not built in)

- 1. Withdrawal Criteria

1. The patient’s refusal to participate in the study or withdrawal of his/her consent to participate in the study
2. Discovery after enrollment that the patient is not eligible for participation in the study
3. Inappropriate for the patient to continue participation in the study due to aggravation of the underlying disease
4. Difficult for the patient to continue participation in the study due to aggravation of a complication
5. Difficult for the patient to continue participation in the study due to adverse event
6. Discontinuation of the entire study
7. Appropriate, in the opinion of the investigator, for the patient to discontinue participation in the study for other reason
8. **Intervention**
   1. Evaluation Schedule

For the present study, evaluation of recurrent AF by garment-type 2-week ECG monitoring and conventional 24-hour ECG monitoring is relevant to this section. Patients will be tested according to the randomized assignment in the period from 3 months post-ablation, when periodic monitoring for AF recurrence is scheduled, following the schedule in the table below.

| **<Conventional Holder ECG first>** | **<hitoe wear ECG first>** |
| --- | --- |

| Item | | Preparation for test | Testing period | | |
| --- | --- | --- | --- | --- | --- |
| Time | | 1 to 3 months before test | Day 0 | Day 14 | Day 15 |
| Informed consent | | ○ |  |  |  |
| Underbust measurement | | ○ |  |  |  |
| Patient enrollment (randomization) | | ○ |  |  |  |
| ECG appointment | | ○ |  | ● | ● |
| Monitoring for adverse event ^a^ | |  |  | ● | ● |
| ECG | Conventional Holter |  |  |  |  |
|  | hitoe wear |  |  |  |  |

| Item | | Preparation for test | Testing period | | |
| --- | --- | --- | --- | --- | --- |
| Time | | 1 to 3 months before test | Day 0 | Day 1 | Day 15 |
| Informed consent | | ○ |  |  |  |
| Underbust measurement | | ○ |  |  |  |
| Patient enrollment (randomization) | | ○ |  |  |  |
| ECG appointment | | ○ |  | ● | ● |
| Monitoring for adverse event ^a^ | |  |  | ● | ● |
| ECG | Conventional Holter |  |  |  |  |
|  | hitoe wear |  |  |  |  |

○ Indicates the items to be performed before ECG start, and ● indicates those to be performed after ECG start.

a: Adverse event is any untoward event, including adverse reaction, regardless of whether it has a causal relationship with the device.

- 1. Contraindications

Do not wear a study device while undergoing magnetic resonance imaging (MRI). Local heat due to induced electromotive force may cause burns.

- 1. Concomitant Use of a Topical Medicine

Use of a topical medicine for skin pruritus will be acceptable.

1. **Efficacy Evaluation**
   1. Efficacy indicator

- Presence of atrial fibrillation lasting for at least 30 seconds
  1. Methods and Timing of Evaluation, Recording, and Analysis

In evaluation of post-ablation AF, the definition of AF recurrence has been established as atrial fibrillation lasting for at least 30 seconds. The acquired ECG data will be automatically analyzed, and the analysis results will be entered in each patient’s electronic medical record. The data will be locked to evaluate the efficacy indicator when data from all 70 patients are accumulated or 6 months after the end of the patient enrollment period, whichever comes earlier.

1. **Safety Evaluation**
   1. Safety indicator

- Presence of skin redness, erosion, or scarring
  1. Methods and Timing of Evaluation, Recording, and Analysis

The safety indicator was set since long-term use of the garment device may cause redness, skin erosion, and rarely scarring at the device contact area in patients with sensitive skin (with a predisposition to allergy) (refer to the package insert). Before removing the study device, a clinical laboratory technologist and a clinical research coordinator (CRC) will conduct a questionnaire (separate sheet) and observe the device contact area, and patients with suspected skin disorder at the device contact area will be seen by a physician.

- 1. Procedures for Information Gathering, Recording, and Reporting on Adverse Events
     1. Preparation of procedures for taking measures in the adverse event

1. The investigator shall prepare procedures for taking actions in the adverse event for each protocol, and act as specified in the procedures.
2. The procedures shall include the flow of reporting by a physician who becomes aware of an occurrence of adverse event, to the investigator or the principal investigator, and seriousness evaluation methods.
3. When matters to be included in the procedures are described in the protocol, there is no need to separately prepare procedures.
   - 1. Actions to be taken in the adverse events

When adverse events occur, the investigator shall discontinue the clinical study or take other necessary actions.

- - 1. Reporting of Adverse Events to the manager of the study site

1. When becoming aware of an occurrence of adverse event (including defects), the investigator shall report to the manager of the study site using the reference form "Report of Adverse Events (including defects)" within the time frame specified in the table "Adverse Events Reportable to the CRB and Reporting Time Frame,” below.
2. The report shall be prepared using the reference form "Report of Adverse Events (including defects)" with attachments prepared using Uniform Form 8, “Report of Adverse Events, regarding Pharmaceuticals," Uniform Form 9, “Report of Adverse Events or Defects regarding Medical Devices," and Uniform Form 10, “Report of Adverse Events or Defects regarding Regenerative Medicine Products, etc." available on the website of the MHLW.
3. When notified by the principal investigator that he/she has reported adverse events (including defects) occurring at another study site in a multicenter study to the CRB, the investigator shall immediately report the information to the manager of his/her study site (see (2)).
   - 1. Reporting of Adverse Events to the CRB (Table 1, Table 2)
4. When becoming aware of an occurrence of adverse events (including defects), the investigator (the principal investigator for the multicenter study) shall report to the CRB within the time frame shown in the corresponding table below.
5. The report shall be prepared using Uniform Form 8, “Report of Adverse Events, regarding Pharmaceuticals," Uniform Form 9, “Report of Adverse Events or Defects regarding Medical Devices," and Uniform Form 10, “Report of Adverse Events or Defects regarding Regenerative Medicine Products, etc." available on the MHLW website, according to the type of report.
6. Even when such information as the cause of the occurrence of adverse event is not available as of reporting, the extent of the information available as of then shall be reported within the specified time frame as the first report. This report shall be immediately followed by a report of details, including the cause, although this reporting does not necessarily have to be completed within the specified time frame.
7. When the CRB provides some advice in regard to the reported content, the investigator shall follow the advice to take necessary actions.
   - 1. Reporting of Adverse Events to the Minister of Health, Labour and Welfare (Table 3)
8. When becoming aware of an occurrence of adverse event, the investigator shall report to the Minister of Health, Labour and Welfare within the time frame shown in the corresponding table below.
9. The report shall be prepared using Attached Form 2-1, “Report of Adverse Events (pharmaceuticals)” and Attached Form 2-2, “Report of Adverse Events (medical devices),” available on the MHLW website.
10. A data entry form available on the MHLW website shall be downloaded and used to prepare a report. A PDF file and an XML file, both created using the entry form, shall be sent by e-mail to the Information Management Division, Office of Safety I, Pharmaceutical and Medical Devices Agency ([trk-shippeitouhokoku@pmda.go.jp](mailto:trk-shippeitouhokoku@pmda.go.jp)).

Table 1 Adverse Events Reportable to the CRB and Reporting Time Frame

| Research category | Reportable events | Reporting time frame |
| --- | --- | --- |
| (ⅰ) Specified clinical research involving unapproved or off-label medicinal products | (1) Any of the following adverse events suspected to be caused by the conduct of the clinical research and unexpected:  ➀ Death  ➁ Life-threatening adverse event | 7 days |
|  | (2) Any of the following adverse events suspected to be caused by the conduct of the clinical research (other than (1)):  ➀ Death  ➁ Life-threatening adverse event | 15 days |
|  | (3) Any of the following adverse events suspected to be caused by the conduct of the clinical research and unexpected (other than (1)):  ➀ Adverse event that requires inpatient hospitalization or prolongation of existing hospitalization for treatment  ➁ Disability  ➂ Adverse event that may lead to disability  ➃ Serious adverse event equivalent to ➀ to ➂ as well as to death, and adverse event that may lead to death  ➄ Congenital disease/anomaly in the subsequent generations | 15 days |
| (ii) Specified clinical research other than (i) | (1) Death (other than that from infection^*1^) suspected to be caused by the conduct of the clinical research | 15 days |
|  | (2) Any of the following adverse events (other than infection^*1^) suspected to be caused by the conduct of the clinical research, and unexpected from the precautions in the package insert or the container/package labels (referred to as “the precautions, etc.”) of the pharmaceuticals, etc. used in the clinical research or expected from the precautions, etc. of the pharmaceuticals but with an unexpected incidence trend or with an incidence trend a change in which may indicate occurrence or spread of health or hygiene hazard:  ➀ Adverse event that requires inpatient hospitalization or prolongation of existing hospitalization for treatment  ➁ Disability  ➂ Adverse event that may lead to death or disability  ➃ Serious adverse event equivalent to death or ➀ to ➂  ➄ Congenital disease/anomaly in the subsequent generations | 15 days |
|  | (3) Adverse event due to infection^*1^ suspected to be caused by the conduct of the clinical research and unexpected from the precautions, etc. of the pharmaceuticals, etc.  (4) Death or (2) ➀ to ➄ due to infection^*1^ suspected to be caused by the conduct of the clinical research (other than (3)) | 1. days |
|  | (5) (2) ➀ to ➄ suspected to be caused by the conduct of the clinical research (other than (2)) | 30 days |
| (ⅲ) Adverse event suspected to be attributable to the conduct of the clinical research: Other than all included in (i) and (ii) | | At the time of periodic report^*2^ |

*1 Infection: Suspected contamination of the pharmaceutical, etc. with a pathogen from a biological material of a biological product, etc.

Testing positive for makers for viruses such as HBV, HCV, and HIV shall also be reported as infection.

*2 At the time of periodic report: At the time of periodic report to the CRB

Table 2 Events of Defects etc. Reportable to the CRB and Reporting Time Frame

| Research category | Reportable events | Reporting time frame |
| --- | --- | --- |
| Clinical research involving medical devices/regenerative medicine products | Defects that may result in the following adverse events: | 30 days |
|  | ➀ Death |  |
|  | ➁ Adverse event that may lead to death |  |
|  | ➂ Adverse event that requires inpatient hospitalization or prolongation of existing hospitalization for treatment |  |
|  | ➃ Disability |  |
|  | ➄ Adverse event that may lead to disability |  |
|  | ➅ Serious adverse event equivalent to ➂ to ➄ as well as to death, and adverse event that may lead to death |  |
|  | ➆ Congenital disease/anomaly in the subsequent generations |  |

Table 3 Adverse Events Reportable to the Minister of Health, Labour and Welfare and Reporting Time Frame

| Research category | Reportable events | Reporting time frame |
| --- | --- | --- |
| Clinical research involving unapproved or off-label medicinal products | (1) Any of the following adverse events suspected to be caused by the conduct of the clinical research and unexpected:  ➀ Death  ➁ Adverse event that may lead to death | 7 days |
|  | (2) Any of the following adverse events suspected to be caused by the conduct of the clinical research and unexpected:  ➀ Adverse event that requires inpatient hospitalization or prolongation of existing hospitalization for treatment  ➁ Disability  ➂ Adverse event that may lead to disability  ➃ Serious adverse event equivalent to ➀ to ➂ as well as to death, and adverse event that may lead to death  ➄ Congenital disease/anomaly in the subsequent generations | 15 days |

- 1. Follow-up Period after Occurrence of Adverse Events

After occurrence of adverse event the patient shall be followed up for approximately 3 months, including observation during outpatient visits.

1. **Statistical Design**
   1. Target Sample Size and Rationale for Setting

It has been shown that the mean daily risk of an AF episode is 6.5% and that the mean 7-day risk of at least one AF episode is 16.7%^5^. Therefore, assuming that the occurrence of AF episodes each day follows a multivariate Bernoulli distribution based on the Gaussian copula and with a conservative estimate of daily AF episode risk of 5.0%, it was then estimated that 17.1% of patients would experience at least one episode in 14 days and that the correlation coefficient between the outcome of at least one episode in 14 days and the outcome of one episode in a day was 0.460. Under these conditions, the minimum sample size that provides a power of at least 0.8 for the primary analysis was determined to be 68 patients, and to allow for dropouts, 70 patients was selected as the sample size.

- 1. Analysis Populations
     1. Efficacy analysis set: Full Analysis Set

All subjects except those who fail to satisfy 1) and 2) in Section 5.1, fail to wear the assigned device after randomization, or have no post-randomization data available

- - 1. Safety analysis set

All subjects except those who have no post-randomization data available

- 1. Analysis Items and Methods
     1. Level of significance and level of confidence

For statistical hypothesis test, a significance level of 0.05 for a two-sided test and 95% confidence interval (CI) will be used.

- - 1. Patient demographics analysis

The frequency or mean and standard deviation will be calculated as appropriate for sex and age.

- - 1. Primary analysis of the primary endpoint

An exact McNemar's test will be performed.

- - 1. Other analyses of the primary endpoint

The percentage and its exact CI will be determined for each device.

- - 1. Secondary endpoint analysis

Binary variables of the secondary endpoints will be analyzed as with the primary endpoint. For frequency variables, basic statistics (mean, standard deviation, median, and interquartile range) will be determined and analyzed by Wilcoxon rank sum test.

- - 1. Safety endpoint analysis

The safety endpoint will be analyzed as with the primary endpoint.

- - 1. Handling of dropouts

In efficacy analysis, test of binary variables will be performed with missing data of dropouts replaced with “absent” for both devices if no data are available for either device, and with “present” for both devices if the result for one device is available and is “present.” For other analyses, no specific processing or imputation or modeling for missing values will be performed.

1. **Source Data Verification**

The investigator and study site shall allow direct access to all study-related records, including source documents, etc., for study-related monitoring and auditing as well as inspections by the CRB and regulatory authorities.

1. **Quality Control and Quality Assurance**

The investigator shall entrust the monitoring and auditing operations to a contract research organization (CRO) to ensure the reliability of the conduct and recording of the study. The investigator and study site shall cooperate with the CRO in monitoring and auditing. The investigator shall inform potential participants in writing that the source documents will be disclosed to monitors and auditors and obtain written consent to such disclosure from the potential participants.

- 1. Monitoring Methods

The monitor shall confirm that the present study is being conducted in compliance with the good clinical research practice. The ICF, case report form (EDC), report of adverse events shall be within the scope of the monitoring. The administrative structure and procedures for monitoring shall be separately defined in written “Procedures for Monitoring.”

- 1. Audit Methods

The auditor shall conduct document-based and on-site inspection independently of monitoring to ensure the reliability of the conduct and recording of the study. As per an auditor’s request, the study staff, etc. must allow direct access to all study-related records. The administrative structure and procedures for auditing shall be separately defined in written “Procedures for Auditing.”

1. **Ethical Considerations: Benefits, Burdens, Expected Disadvantage**
   1. Human Right Considerations (personal information protection, prohibition of unintended use, etc.)
2. Raw data on the study conduct and ICF, etc. shall be handled with due consideration given to confidentiality of patients. Any reports to be submitted outside of the study site shall be anonymized using patient identification code, etc. Publication of the study results shall include no patient-identifying information.
3. If patient samples, etc. are transferred outside of the study site for measurements, etc., methods for anonymization, storage, and disposal, and the scope of persons with access to sample information shall be defined in advance.
4. Patient data obtained in association with the study shall not be used for any purpose other than the study. Any secondary use in the future shall be limited to the data of patients who provide written consent to such use.
   1. Safety/Disadvantage Considerations
      1. The countermeasures to possible risks described in Section 4.4.2, "Expected adverse reactions and expected defects," shall be considered to minimize the risks.
      2. In the adverse events, the patient shall be appropriately examined and treated immediately.
      3. Information necessary for safe conduct of the clinical study shall be collected, and if necessary, the protocol shall be amended.
      4. If "the criteria for study discontinuation/interruption" are satisfied, appropriateness of continued study shall be considered.
5. **Record Management**
   1. Collection and Handling of Samples, etc.

Source documents shall be retained at the study site for 10 years after completion of the study and destroyed according to the internal regulations. No samples, etc. will be collected.

- 1. Description of Provision of Samples/Data, etc.
- The ICFs shall be retained at University of Tsukuba (source) for at least 3 years after completion of provision of the items shown below.
- The protocol shall be retained at University of Tsukuba (source) for at least 5 years after completion of the study.
- Name of the destination institution: Toray Industries, Inc.
- Name of the study director at the destination institution: Hirokazu Sugihara
- Name of the source institution: University of Tsukuba Hospital
- Name of the study director at the source institution: Takeshi Machino
- Sample/data items to be provided: ECG data, medical records
- Process for obtaining samples/data: Obtained during the course of this study at University of Tsukuba Hospital
- Address of the source institution: 2-1-1 Amakubo, Tsukuba, Ibaraki, Japan
- Name of the head of the source institution: Akira Hara

1. **Payments and Compensations**
   1. Compensation for Injury

As the present study involves no invasive procedures and does not exceed the scope of routine medical care, there is no need to enroll in clinical research insurance. Any injury resulting from participating in the present study will be treated using health insurance.

- 1. Enrollment in Liability Insurance

The investigator will enroll in the medical professional liability insurance against damages.

- 1. Patient Payment (and honorarium, etc.)

As Holter ECG performed in the present study is within the scope of routine medical care, patients will have to pay a copayment with the health insurance coverage. The expenses for the 2-week ECG monitoring with the hitoe^®^ wear in the present study will be paid using the research funding under the joint research agreement with Toray. To reduce patient burden, QUO cards (10,000 yen/person) will be distributed at the completion of participation.

1. **Publication of Information on Clinical Study**
   1. Registration of Research Overview and Results

The clinical study shall be registered and published in the jRCT (Japan Registry of Clinical Trials) by means of notifications and reporting to the Minister of Health, Labour and Welfare.

- 1. Publication of Study Results

The study results are planned to be published in paper presentations, conference presentations, and on the internet. Also, the results may be presented in a press release of the joint research partner Toray as part of its new product public relations activities.

1. **Study Period**

The study period is scheduled to be August 1, 2018 through December 31, 2020 (enrollment deadline: December 13, 2019).

1. **Informed Consent**
2. Potential participants shall be fully informed orally and in writing using the written information for patients approved by the CRB, and shall provide written consent to participate in the study of their own free will. Consent shall not be obtained from minors or legally acceptable representatives of potential participants.
3. The written information for patients shall be updated immediately if any new information that may affect a participant's willingness to continue to participate in the study becomes available.
4. The ICF shall be understandable to participants, and the versions shall be appropriately managed.
5. The ICF format shown in a separate sheet shall be followed.
6. **Requirements for Proper Conduct of Clinical Study**
   1. Research Funds and Conflicts of Interest for Research Institutions

Under a joint research agreement, this study will be funded (and supplied with the study devices) by Toray Industries, Inc. and the investigational devices to be used in the study will be lent free of charge by its marketing authorization holder, Toray Medical, Co., Ltd. Toray Industries, Inc. will cooperate in preparing the protocol, but shall not be engaged in the conduction, analysis, or reporting of the study. The present study shall be subject to monitoring and audit.

- 1. Conflicts of Interest for Research Staff

Some of the sub-investigators belong to empowered courses sponsored by the companies, etc. associated with the present study or are paid for providing those companies, etc. with consulting services; however, in accordance with the Conflict of Interest Management Standards, such persons shall not be engaged in tasks related to data management, monitoring, or statistics/analysis.

- 1. Update of Disclosures

At the beginning of each fiscal year, to ensure that the conduct of the study will not impair the rights and interests of patients, the investigator shall confirm with the study staff that there are no new "conflicts of interest" that may affect the study results or interpretation of the results in the design, conduct, or reporting of the present study.

1. **List of References/Literature**
2. Pokushalov E, et al. Ablation of paroxysmal and persistent atrial fibrillation:1-year follow-up through continuous subcutaneous monitoring. J Cardiovasc Electrophysiol. 2011; 22: 369-375.
3. Calkins H, et al. 2017 HRS/EHRA/ECAS/APHRS/SOLAECE Expert Consensus Statement on Catheter and Surgical Ablation of Atrial Fibrillation. Heart Rhythm. 2017; 14: e275-e444.
4. Kawasaki S, et al. Recurrence of atrial fibrillation within three months after pulmonary vein isolation for patients with paroxysmal atrial fibrillation: Analysis using external loop recorder with auto-trigger function. J Arrhythmia. 2015; 31: 88-93.
5. Haruki Y, et al. Evaluation for the recurrence of atrial fibrillation after radiofrequency catheter ablation with use of an external loop recorder. Japanese Journal of Electrocardiology. 2017; 37: 5-11 [in Japanese].
6. Herskind M, Nielsen J, Damgaard D, Sandal B. Atrial fibrillation detected by external loop recording for seven days or two-day simultaneous Holter recording: A comparison in patients with ischemic stroke or transient ischemic attack. J Electrocardiol. 2017; 50: 287-293.
7. **Appendices (package inserts)**

See separate sheets.
